# Does a 4 diagram manual enable laypersons to operate the laryngeal mask supreme^®^? A pilot study in the manikin

**DOI:** 10.1186/1757-7241-20-21

**Published:** 2012-03-27

**Authors:** Gereon Schälte, Christian Stoppe, Rolf Rossaint, Laura Gilles, Maike Heuser, Steffen Rex, Mark Coburn, Norbert Zoremba, Annette Rieg

**Affiliations:** 1 Department of Anesthesiology, University Hospital Aachen, Aachen, Germany; 2 Department of Anesthesiology, University Hospital Leuven, Leuven, Belgium; 3 Department of Anesthesiology, University Hospital Aachen, Pauwelsstr. 30, 52074 Aachen, Germany

## Abstract

**Background:**

Bystander resuscitation plays an important role in lifesaving cardiopulmonary resuscitation (CPR). A significant reduction in the "no-flow-time", quantitatively better chest compressions and an improved quality of ventilation can be demonstrated during CPR using supraglottic airway devices (SADs). Previous studies have demonstrated the ability of inexperienced persons to operate SADs after brief instruction. The aim of this pilot study was to determine whether an instruction manual consisting of four diagrams enables laypersons to operate a Laryngeal Mask Supreme^® ^(LMAS) in the manikin.

**Methods:**

An instruction manual of four illustrations with speech bubbles displaying the correct use of the LMAS was designed. Laypersons were handed a bag containing a LMAS, a bag mask valve device (BMV), a syringe prefilled with air and the instruction sheet, and were asked to perform and ventilate the manikin as displayed. Time to ventilation was recorded and degree of success evaluated.

**Results:**

A total of 150 laypersons took part. Overall 145 participants (96.7%) inserted the LMAS in the manikin in the right direction. The device was inserted inverted or twisted in 13 (8.7%) attempts. Eight (5.3%) individuals recognized this and corrected the position. Within the first 2 minutes 119 (79.3%) applicants were able to insert the LMAS and provide tidal volumes greater than 150 ml (estimated dead space). Time to insertion and first ventilation was 83.2 ± 29 s. No significant difference related to previous BLS training (P = 0.85), technical education (P = 0.07) or gender could be demonstrated (P = 0.25).

**Conclusion:**

In manikin laypersons could insert LMAS in the correct direction after onsite instruction by a simple manual with a high success rate. This indicates some basic procedural understanding and intellectual transfer in principle. Operating errors (n = 91) were frequently not recognized and corrected (n = 77). Improvements in labeling and the quality of instructional photographs may reduce individual error and may optimize understanding.

## Introduction

Layperson resuscitation plays an important role in providing lifesaving cardiopulmonary resuscitation (CPR) and bridging the interval to the arrival of healthcare professionals. However, only 50% of laypersons actually administer "first aid" in such situations [1,2]. Reasons given are various and include a lacking sense of personal responsibility when there are many other people "on site", an aversion to strangers' bodily fluids and the percieved risk of infection during "mouth to mouth" ventilation. Individuals may be discouraged from administering CPR by a lack of confidence in their abilities and a fear of doing more harm than good [3-5].

Immediate resuscitation by laypersons and early defibrillation have been identified as key factors positively influencing clinical outcome, survival, and costs [6-8]. In consequence, chest compressions, securing of the airway, initiating (mouth-to-mouth) ventilation, and the use of an automated defibrillator have been implemented in the basic life support guidelines [9]. In the further course of (layperson) CPR securing adequate ventilation is of paramount importance for a full recovery. Mask ventilation requires considerable skill and is associated with the risk of stomach inflation, regurgitation and aspiration. Therefore, it is only recommended for emergency professionals [10]. During CPR a significant reduction in the "no-flow-time", quantitatively better chest compressions and an improved quality of ventilation can be demonstrated using supraglottic airway devices (SADs) [11,12]. Moreover, unsecured airways during CPR lead to further pulmonary complications. Aspiration is frequently evident in unconscious patients on arrival by ambulance [13-15]. SADs are shown to reduce the incidence of aspiration compared to bag valve mask device ventilation during CPR [16].

Endotracheal intubation (ETI) remains the standard in pre-hospital airway management, where experienced personnel are present. However, there is controversy as to whether ETI is really the best option for patients when it is performed by paramedics or inexperienced physicians. In these cases, as well as in patients in whom prolonged or multiple intubation attempts are needed, SADs may be the better alternative [17]. SADs have been shown to be easily inserted regardless of level of experience, and properly operated in the manikin as well as in patients in both clinical and preclinical settings [11,12,18].

Various training approaches have been investigated. These vary from the traditional (educational session, manikin training and/or practice training, practice) to more compact and minimalistic concepts (i.e. instruction by telephone, educational movie, 3 min demonstration) [19-22].

The aim of this study was to determine whether a short instruction manual consisting of four diagrams only enables laypersons to operate a Laryngeal Mask Supreme^® ^(LMAS) in a manikin, as well as to analyze potential pitfalls and the transfer of given information.

## Methods

The institutional review board waived the requirement for written informed consent, as no potential harm to the study participants was involved. No personal data except age, academic affiliation and prior "first-aid" knowledge was collected. All subjects agreed to be evaluated anonymously for scientific and educational purposes. Prerequisites for inclusion were the lack of any previous medical education (i.e. physician, nurse, paramedic) other than a BLS course, and an age of 18 or older. Applicants were recruited at the RWTH Aachen University (**R**heinisch-**W**estfälische-**T**echnische-**H**ochschule Aachen) campus (Audimax & Kármán Auditorium). Experimental data were recorded "on-site" in February and March 2011.

A resuscitation scenario with a manikin (Ambu M MegaCode W^®^, Ambu GmbH, Bad Nauheim, Germany) was prepared. The experimental scene was separated by partition walls.

**Figure 1:**
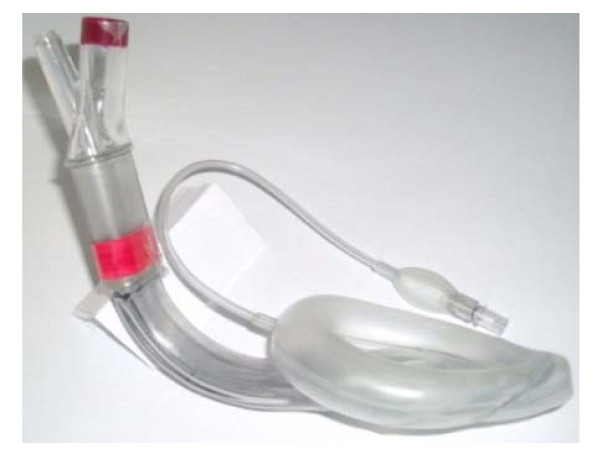
**Laryngeal mask Supreme^® ^and indicator labels**. BMV ISO connector and estimated depth of insertion (mouth) were labeled red.

**Figure 2:**
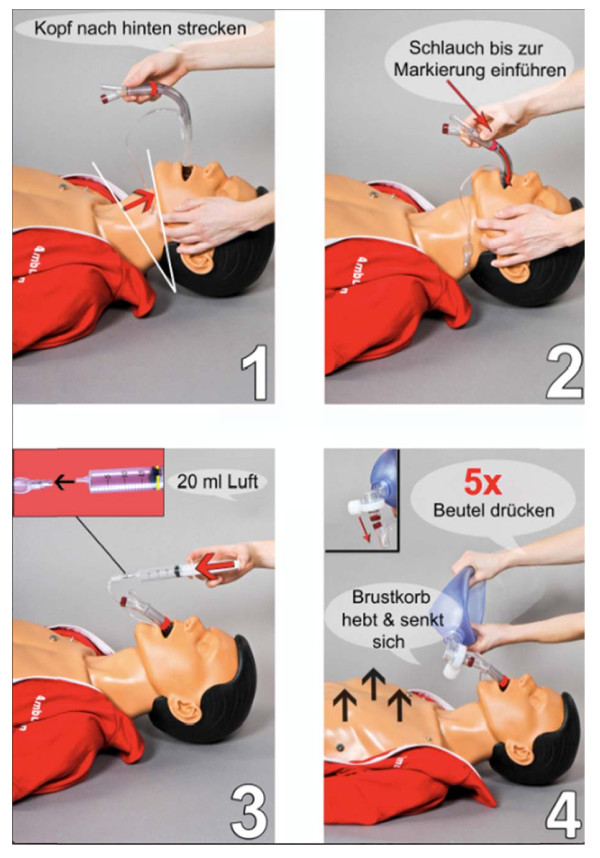
**Instruction sheet**. Four diagram instruction manual. Essential steps of insertion are presented in chronologic order (1-4) and manual maneuvers highlighted with red arrows and "close-ups". Key commands were shown as speech balloons. Picture 1: "recline the head"; Picture 2: "insert the device up to the indicator label"; Picture 3: 20 ml of air; Picture 4: compress the bag valve 5 times; chest will rise and fall.

A technical step-by-step instruction manual for the suggested use of the LMAS, inflation syringe and BMV had been prepared. Pictures illustrating the key actions and the suggested correct use of the devices were prepared and displayed on an instruction sheet (Figure 1). For simplicity and to aid recognition the LMAS and the BMV connectors were labeled red. A further mark was added to the laryngeal mask labeling roughly the correct depth of insertion (Figure 2). The cuff inflation syringe was prepared with a fixed volume of 20 ml air. All devices were packaged in a bag together with the instruction sheet. Squeezing the BMV was displayed using two hands. The LMAS (size 4) was established to fit to the manikin's anatomy and to provide an adequate seal after the cuff has been inflated with 20 ml air.

Applicants were approached personally and asked to participate in a scientific trial investigating a new alternative to "mouth-to-mouth" ventilation in a dummy and, after agreement, were handed a standardized instruction sheet:

*"Behind the wall you will find an unconscious person. This person has stopped breathing. You are responsible for their VENTILATION. In this setting you should use the BOXED DEVICES next to the head. Do not perform "mouth-to-mouth" ventilation. Open the bag and proceed as displayed on the instruction sheet"*.

Participants then entered the experimental scene and proceeded. Time was recorded starting when the bag was opened and stopped either after ventilation was correctly initiated or the trial was ended by the applicant or - after 4 min - by the investigators. The correct insertion of the LMAS, cuff inflation, connection and compression of the BMV were judged. Multiple compressions of the BMV were allowed as displayed in the manual. In addition, we recorded the number of insertions completed within 2 minutes of the start of the trial. After the trial applicants were interviewed and asked their opinions of the materials, instructions, and their understanding of BMV ventilation. Moreover, they were asked whether they believe that following this instruction sheet and this single trial they would be able to operate the LMAS in a real resuscitation. A tidal volume of > 500 ml for ventilation was judged as sufficient according to the ERC guidelines. Tidal volumes of between 150 ml and 500 ml were judged as "ERC insufficient" but ventilation and tidal volumes < 150 ml were judged as insufficient.

### Statistics

Statistical analysis was performed using SAS (Statistical Analyses System), (SAS Institute GmbH, Heidelberg, Germany). A success rate of 95% was expected [19,21]. The power of the study was calculated with a significance level, α = 0.05. A power of 80% results in a sample size of 120. In total 150 study subjects were included to compensate for possible dropouts. The power calculation was performed using nQuery Advisor^® ^Version 7.0 (Statistical Solutions, Saugus, MA, USA).

A Chi-square test was used to calculate statistical differences in success rate with respect to gender, previous BLS training, and studying in the field of engineering. *T*-test was used to calculate statistical differences between time of insertion and age or sex. Correlation was calculated by regression analysis. Data are presented as means ± standard deviation until stated otherwise. A P < 0.05 indicated statistical significance.

## Results

**Figure 3:**
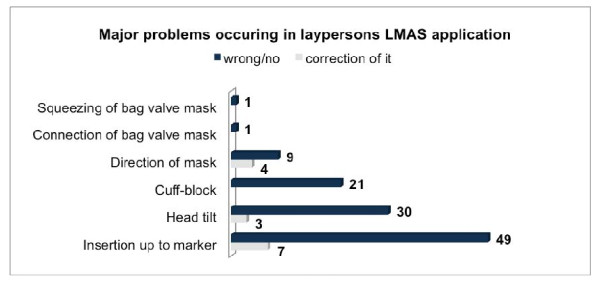
**Number and origin of errors and individual correction**. Data are numbers.

Data from 150 participants (121 male, 29 female) were analyzed. Mean age was 22.9 years (22.9 ± 2.8). Overall 145 participants (96.7%) inserted the LMAS in the correct direction in the manikin. Within a 2 min period 119 (79.3%) applicants were able to insert the LMAS and provide tidal volumes greater than 150 ml (approximate dead space). 74% (n = 111) of the participants were able to deliver tidal volumes of greater than 500 ml during these first 2 minutes. The device was inserted inversely or twisted in 13 (8.7%) attempts. 8 (5.3%) individuals recognized and corrected the position. The most common fault was an incorrect depth of insertion (n = 61; 40.7%). 20 applicants successfully corrected the depth to the labeled level. The second most common fault was the tilt of the manikin's head (n = 30; 20%) and this made ventilation impossible in 5 cases. In 21 cases (14%) the problem concerned the cuff, i.e. cuff inflation was insufficient (n = 15) or simply omitted (n = 6). In one trial each, the connection of the BMV (0.67%) or squeezing the BMV were omitted (0.67%) (Figure 3). In total the insertion of the LMAS was performed "correctly" by 82 participants (55%) on the first attempt. Despite an obviously sufficient insertion in 18 (12%) attempts no ventilation could be detected and was associated with forgotten or inadequate cuff inflation in 9 efforts. Overall a total of 91 operating errors of different severity and impact could be identified. Only 14 (15.4%) were corrected. The time from start of insertion to first ventilation was 83.2 ± 29 s.

**Figure 4:**
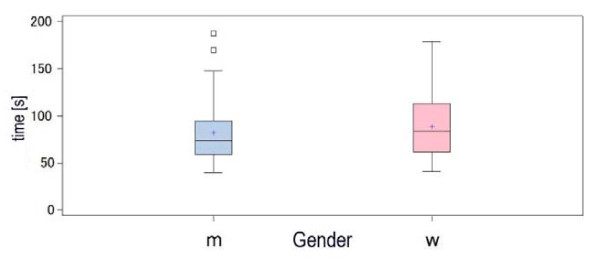
**Time to insertion and gender**. No significant difference was found between genders in time to insertion. Data are mean ± SD.

No gender related difference could be observed regarding the time to successful ventilation (male 81.8 ± 28.24 vs female 88.8 ± 32.11 s; P = 0.25) (Figure 4).

**Figure 5:**
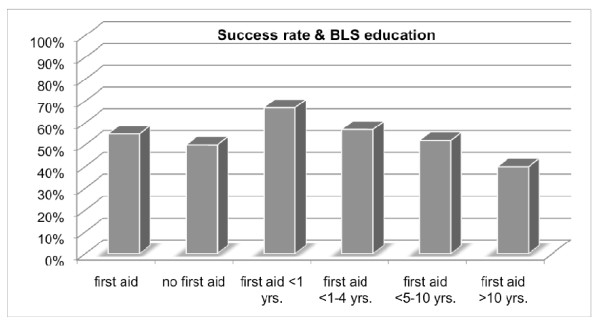
**Rate of success and previous BLS education**. Data are presented as percentages. No significant difference was found between. BLS providers and participants without BLS training (P = 0.84).

Most of the participants had prior training in BLS (92%). Time passed since the BLS training was 1-4 years in 52% and 5-10 years in 38% respectively. In 7% training had taken place less than one year ago, and in 4% more than 10 years ago. No significant difference in performance related to previous BLS training was observed (P = 0.85) (Figure 5).

**Figure 6:**
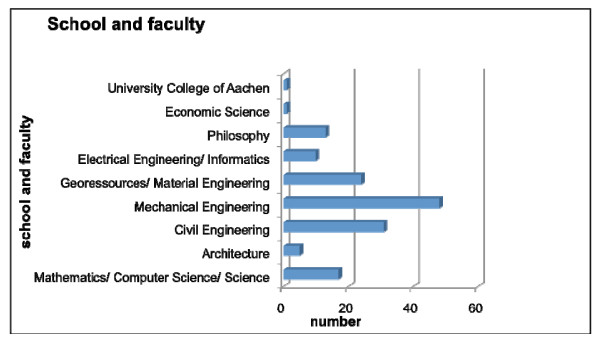
**Participant's classification with regard to school and faculty**. Data are total numbers.

77.7% of the male and 41.4% of the female applicants studied in the field of "engineering". (Figure 6). No statistically significant differences between the "engineers" and the other faculties regarding "success rate" could be shown (P = 0.07).

23% of applicants complained about problems related to their understanding of the instruction sheet.

A total of 63% of participants reported to be confident in using the LMAS in a real life resuscitation. 33% thought that they would become confident in operating the LMAS after further training, and only 2% stated they would decline the use of LMAS as lay responders.

## Discussion

In this pilot study we evaluated a simple 4-picture instruction manual designed to enable laypersons to operate the LMAS during BLS without any further verbal guidance or "hands-on" training. Overall laypersons were able operate the LMAS with a success rate of 79.3% in the manikin.

This is one of the few trials investigating "genuine" laypersons and their performance operating an SAD "on-site" without any prior training [19]. We focus on specifics of their performance, and expose the pitfalls and potential lack of clarity when transferring pictures into action.

Most of the previous trials published have involved medical, paramedical, nursing students and junior doctors, designating them as "lay persons" [23-28]. Thus, bias resulting from a general interest in the field as well as related medical procedural knowledge cannot be ruled out.

Traditional CPR training is usually in the form of an instructor directed demonstration, frequently followed by brief "hands-on" exercises. Success rates inserting SADs were shown to be as high as > 90% in the manikin and > 80% in patients [23,26,29,30]. As a result, over the past years alternative methods of instruction using newer more media based approaches have become a major topic of research. After scripted telephone instructions, a brief demonstration only, or a video-clip demonstration, success rates of 80% - 95% have been demonstrated [19-21]. In this trial using a four diagram illustrated instruction manual we show that 96.7% of the 150 applicants inserted the LMAS in the right direction. This implicates at least a basically correct transfer of information provided in the diagrams. However, in the 13 participants inserting the device rotated, only 8 recognized and corrected the position. Improved labeling (e.g. similar colors or legible print) and more "close-up" pictures might lead to better understanding. Additional verbal instructions might have solved these problems [22].

We found the depth of insertion one of the major problems in operating the LMAS in this context. 61 of the LMAS (40.7%) were not inserted at the correct depth as indicated. After recognizing the problem and correcting it, 41 LMAS (27%) remained malpositioned with regard to depth. This might be explained by two factors. First, in the manikin and despite lubrication, friction between two synthetic materials may create the need for more force than is expected by participants. Despite close resemblances even high-fidelity manikins are unable to recreate the feel and finer aspects of human airway anatomy [31]. Second, the landmarks and system of labels chosen are not sufficiently intuitive for laypersons. Nevertheless in 29 of the remaining 41 LMAS inserted not deeply enough ventilation could be provided, though this may possibly be related to characteristics of the Manikin itself. Failing to tilt the manikin's head was the second most obvious mistake made during individual trials (20%). In the 5 cases of impossible ventilation the manikin's head was tilted in anteflexion and not positioned neutral or in retroflexion. In general, insertion of SADs is recommended in a neutral or mildly reclined position but does not require a fully reclined head [29,30,32].

Problems with the cuff or its inflation, i.e. an incomplete or omitted inflation, were noted during 21 individual trials (and resulted to some extent in insufficient ventilation (n = 9) of the manikin). None of the participants recognized or corrected this problem. Kurola et al. recently identified cuff related problems as the major hurdles in operating the laryngeal tube following brief written instructions [22]. The authors concluded that special attention should be given to avoiding improper cuff inflation and to the proper depth of insertion, and recommend training by a professional instructor.

Alternatives worth discussing are a self-expanding cuff or an SAD without an inflatable cuff, e.g. the I-Gel laryngeal mask (I-Gel) [25,33-37]. Eliminating the need to first connect a syringe and then inflate the cuff would eliminate two potential sources of error and thus improve time to ventilation.

Female and male participants performed equally regarding time to successful ventilation in this trial. The prevalence of BLS training in women is hypothesized to be lower in the literature [36]. In this trial no differences between BLS education and gender could be observed.

The time from start of the scenario to definitive ventilation (one measure of "performance") should be interpreted with caution. In comparison to recent trials investigating different training modalities (traditional, verbal, video, telephone or combined), in our trial the time to ventilation was prolonged [19-22]. Participants were instructed "on-site" and needed time to read the visual instructions and identify the devices before taking action. A mean of 83 s seems rather long in this context and differs up to 55 s from the time to ventilation when instructions were given before the start of the trial by which time substantial learning has already occurred [22]. Nevertheless, this is the new approach tested in this study. The starting of resuscitation within the first 2 or 3 minutes following cardiac arrest is compatible with good neurological outcomes, hence this approach may be a step toward better compliance of laypersons with the demand for bystander resuscitation. "On-site" instruction using supraglottic airways may be a useful tool in layperson CPR in multi-bystander scenarios or whenever one of the bystanders is familiar with operating SADs and may correct or improve the initial operator's attempt at insertion [22].

The RWTH Aachen University was founded as a technical university, with a majority of male students. Schools and faculties of technical and economical engineering still dominate. In this historical context the allocation of participants and the majority of male participants can be explained. We demonstrate that being an engineer, implying a more developed level of technical understanding, did not correlate with a higher success rate compared to non- engineering faculties.

Some limitations need to be discussed. Results presented are obtained in a manikin model and cannot be simply transferred to the patient. The time to insert an SAD in a manikin can be significantly different from that in a patient [31]. Moreover, insertion of an SAD and obtaining a seal is shown to be significantly more difficult in patients than in manikins and is associated with lower success rates and a longer time of insertion [26]. Accordingly we simply note our combined endpoint "time to insertion and successful ventilation" and refrain from further discussion in favor of presenting our procedural findings. One point should be emphasized. This is a feasibility study investigating the efficacy of a standardized instruction manual, and any potential errors transferred into individual practice. Therefore a standardized environment with robust materials - as provided by the manikin - is required [31]. It is well known that no one manikin performs best for SAD insertion. Studying the performance of SADs therefore requires careful selection [38]. Finally, by choosing students we are focused on a highly educated group compared to the general population. It remains speculative as to whether groups of different levels of education would perform differently.

In the manikin retention of skills in operating SADs were shown to be high [18,24,39,40]. Having first aid training increased the likelihood of intervention and of owning a first aid kit or pocket mask [41].

It remains speculative whether, if supraglottic airway devices were implemented in BLS training, after a certain period of time without training, an illustrated manual may improve laypersons' performance or their willingness to provide (advanced) airway management.

## Conclusion

In this pilot study 119 (79.3%) laypersons were able to insert the LMAS and provide tidal volumes greater than 150 ml in the manikin with "on-site" illustrated instructions. Visualizing and understanding the procedure, i.e. inflating the cuff and inserting the LMAS deep enough and in the right direction were identified as the major obstacles. Most faults could have been verbally corrected leading to an even higher success rate. Future improvements in labeling and in the quality of the diagrams may lead to faster and better performance. These findings support the implementation of SADs in BLS training analogously to the implementation of AEDs.

## Competing interests

The authors declare that they have no competing interests.

## Authors' contributions

All authors have read and approved the final manuscript. GS, CS, RR and AR designed the study. GS, CS, LG, MH and NZ allocated data and performed the trial. GS, MC and SR performed the statistical analysis. GS, CS, RR and AR drafted the manuscript. MC, SR and RR critically revised the manuscript.
